# Growth Differentiation Factor 15 Protects SH-SY5Y Cells From Rotenone-Induced Toxicity by Suppressing Mitochondrial Apoptosis

**DOI:** 10.3389/fnagi.2022.869558

**Published:** 2022-06-02

**Authors:** Peizheng Li, Hongbo Lv, Bohan Zhang, Ruonan Duan, Xiufang Zhang, Pengfei Lin, Chengyuan Song, Yiming Liu

**Affiliations:** ^1^Department of Neurology, Qilu Hospital, Shandong University, Jinan, China; ^2^Department of Neurology, Tianjin Medical University General Hospital, Tianjin, China

**Keywords:** Parkinson’s disease, GDF15, mitochondria, apoptosis, p53, neurodegeneration

## Abstract

**Objective:**

Parkinson’s disease (PD) is the second most common neurodegenerative disorder worldwide. Mitochondrial dysfunction is suspected as one of the pathogenic mechanisms of PD. Growth/differentiation Factor-15 (GDF15) has been reported to affect mitochondrial function in PD. However, the relationship between mitochondrial function and GDF15 induction has not been explained well. Hence, we aimed to reveal the effect of GDF15 induction on SH-SY5Y cells with rotenone toxicity, a cell model of PD.

**Methods:**

SH-SY5Y cells were exposed to 1 μM rotenone as a PD model. Cells were transfected with a GDF15-overexpression plasmid and empty vector. We then analyzed the expression level of GDF15, BCL-2/BAX, P53, PGC1-α, α-syn, and TH in GDF15-overexpressing cells by western blotting, enzyme-linked immunosorbent assay, and quantitative real-time polymerase chain reaction. The cytotoxicity of rotenone was measured by CCK-8 assays. Cell apoptosis was detected by flow cytometric and TUNEL assays. The effect of GDF15 on oxidative stress and mitochondrial function was revealed using DCFH-DA, mito-SOX, and JC-10 assays and a Seahorse XF Cell Mito Stress Test.

**Results:**

GDF15 protected rotenone-treated SH-SY5Y cells from toxicity by preserving mitochondrial function and decreasing apoptosis, during which GDF15 might function by influencing PGC1α through the regulation of p53. In addition, GDF15 overexpression could improve Akt and mTOR phosphorylation, leading to PI3K/Akt/mTOR pathway activation. However, these protective effects were eliminated when cells were treated with the PI3K/Akt specific inhibitor LY294002.

**Conclusion:**

Our findings suggest that GDF15 can protect mitochondrial function and inhibit apoptosis in SH-SY5Y cells after exposure to rotenone by upregulating PGC1α *via* p53. These properties might comprise its anti-apoptotic effects, mediated by the PI3K/Akt/mTOR signaling pathway.

## Introduction

Parkinson’s disease (PD) is a common disease estimated to affect approximately 61 million people worldwide 2016 (Dorsey et al., 2018a). For reasons that are still not well understood, the incidence and prevalence of PD has increased rapidly over the past two decades (Dorsey et al., 2018b; Deuschl et al., 2020; Bloem et al., 2021). PD has a huge effect on the individual, with typical manifestations such as bradykinesia, tremors, and rigidity. Furthermore, the disease deeply affects caregivers and causes an excessive and developing burden for society (Dorsey et al., 2018a).

Although the exact cause of PD is not yet understood, it has been suggested that factors causing mitochondrial dysfunction have a crucial role in its pathogenesis (Hauser and Hastings, 2013). Growth/differentiation factor-15 (GDF15, also known as macrophage inhibitory cytokine-1, MIC1), is a newly discovered member of the transforming growth factor β (TGF-β) superfamily. It is a widespread pleiotropic cytokine that plays a role in a variety of pathologies, including inflammation. It has also been found to be an autocrine regulator of macrophage activation and a target of several cytokines, including interleukin-1 beta (IL-1β), tumor necrosis factor-alpha (TNF-α), interleukin-2 (IL-2), and transforming growth factor-beta (TGF-β) (Bootcov et al., 1997). In the last two decades, GDF15 has attracted significant attention in biological and medical fields owing to its multiple roles in a variety of diseases, including cardiovascular disease and cancer (Hong et al., 2014; Mehta et al., 2014; Wang et al., 2014; Liu et al., 2015). Recently, serum GDF15 levels have been associated with mitochondrial diseases (Koene et al., 2015; Yatsuga et al., 2015). Moreover, GDF15 has been identified as a potential diagnostic biomarker for neurodegenerative diseases such as PD (Maetzler et al., 2016; Miyaue et al., 2020). Our previous study also demonstrated that the serum GDF15 levels are higher in PD patients than in healthy individuals (Yao et al., 2017). In addition, GDF15 can regulate biological effects by modulating the signal cascade activated by other differentiation or growth factors. However, findings that question whether GDF15 is related to PD have also been reported (Davis et al., 2020). Thus, the physiological effects of endogenous GDF15 and the associated molecular mechanism with respect to the appearance and development of neurological diseases must be further investigated.

The SH-SY5Y cell line, a three-times cloned subline of a cell line from a neuroblastoma patient with sympathetic adrenergic ganglion origin, has been used widely in experimental neurological studies, including analyses of neuronal metabolism, differentiation, neurodegenerative disease. In this study, we sought to establish a direct relationship between mitochondrial function and GDF15 induction in a cell model of PD with the SH-SY5Y cell line exposed to rotenone, a broadly used cell model to mimic the pathogenesis of PD (Johnson and Bobrovskaya, 2015). In addition, we investigated the anti-apoptotic and anti-inflammatory effects of GDF15 in rotenone-exposed cells and determined whether GDF15 elevates mitochondrial functions in these cells. Therefore, this study provides a range of pathophysiological insights into PD and indicates that GDF15 is a potentially promising therapeutic agent for the treatment of parkinsonism.

## Materials and Methods

### SH-SY5Y Cell Culture and Reagent Treatment

The SH-SY5Y cell line, which was produced by the American Type Culture Collection (ATCC, Manassas, VA, United States), was cultured in Dulbecco’s modified Eagle’s high glucose medium (DMEM, 11965, Life Technologies, Rockville, MD, United States), supplemented with 10% fetal bovine serum (10099, Invitrogen, CA, United States) in a humidified incubator with 5% CO_2_ at 37°C. We prepared rotenone (R8875, Sigma-Aldrich, St Louis, MO, United States) in dimethyl sulfoxide (DMSO; D2650, Sigma-Aldrich) at a stock concentration of 50 mM. It was then diluted with DMEM to a concentration of 1 μM and incubated with cells for different amounts of time (0, 12, 24, 36, and 48 h) according to the corresponding experiments. The vehicle was used as the control. The PI3K/Akt specific inhibitor LY294002 (A8250, APExBIO, Houston, TX, United States) was diluted with DMEM to a concentration of 0–10 μM and incubated with cells for 3 h to choose the appropriate stimulus concentration.

### GDF15-Overexpression Plasmid Transfection

GDF15 was overexpressed by transfecting cells with pcDNA3.1-GDF15. cDNA encoding full-length *GDF15* was cloned into the pcDNA vector, and the sequence was confirmed using Sanger sequencing. Empty plasmids were used as the mock control. All plasmids were verified using DNA sequencing. Lipofectamine 2000 (11668, Invitrogen, Waltham, MA, United States) was used for cell transfections following the manufacturer’s instructions.

### Measurement of Human GDF15 Levels in the Cell Culture Supernatant

The cell culture supernatant was collected after 0–48 h of treatment with rotenone (1 μM) and stored at –80°C until analysis. GDF15 concentrations were measured using a high-sensitivity human enzyme-linked immunosorbent assay (ELISA) kit (ab155432, Abcam, Cambridge, MA, United States) as per the manufacturer’s instruction.

### Western Blotting Analysis

Levels of protein expression were measured using western blotting analysis, which was performed using standard methods with commercially available antibodies. After treating cells with vehicles or 1 μM rotenone for 24 h, the whole-cell lysate was prepared using lysis buffer, protease inhibitors, and phenylmethylsulphonyl fluoride. The protein concentration was measured using a Bicinchoninic Acid protein assay kit (23227, Thermo Scientific, Waltham, MA, United States). Soluble proteins (30 μg) were separated using 8–12% SDS-polyacrylamide gels and then transferred onto polyvinylidene difluoride membranes (200 mA, 60 min), which were then blocked in 5% non-fat milk or bovine serum albumin in tris-buffered saline with tween 20 and incubated with primary antibodies overnight. The primary antibodies used were as follows: anti-GDF15 (1:5000, ab39999, Abcam), anti-Bcl-2 (1:2000, ab182858, Abcam), anti-Bax (1:1000, ab182734, Abcam), anti-p53 (1:1000, ab32049, Abcam), anti-PGC-1α (1:1000, ab106814, Abcam), anti- tyrosine hydrolase (TH) (1:200, ab112, Abcam), anti-mTOR (1:1000, 2983S, Cell Signaling Technology, Danvers, MA, United States), anti-phospho-mTOR (1:1000, 5536S, Cell Signaling Technology), anti-Akt (1:1000, 4685S, Cell Signaling Technology), anti-phospho-Akt (1:1000, 4060S, Cell Signaling Technology), and anti-β-actin (1:5000, BE3212-100, EASYBIO, Seoul, South Korea), used as an internal control. Then, secondary HRP-linked antibodies were used to detect primary antibodies. The immunoreactive signal was detected using an ECL substrate kit (Millipore, Billerica, MA, United States). Gray values of the protein bands were analyzed using ImageJ 1.8.0r software (National Institutes of Health, Bethesda, MD, United States).

### RNA Isolation and Quantitative Real-Time Polymerase Chain Reaction

RNA was isolated from cells following the manufacturer’s protocol using TRIzol (Invitrogen, 15596-026), and reverse transcription was used to convert 1 μg RNA to cDNA using a cDNA Synthesis kit (Vazyme, R323-01). Quantitative real-time polymerase chain reaction (qRT-PCR) analysis was performed using the Super Real PreMix Plus SYBR Green (Vazyme, Q711-02). The relative mRNA expression levels were normalized to those of *GAPDH* and calculated using the 2^–ΔΔCT^ method. The primer sequences for qRT-PCR were as follows: PPAR-γ coactivator-1-alpha (PGC1-α): forward: 5′-TCCTCACACCAAACCCACAG-3′ and reverse: 5′-TGGGGTCATTTGGTGACTCTG-3′, α-synuclein: forward: 5′-AATGAAGAAGGAGCCCCACAG-3′ and reverse: 5′-AGGCTTCAGGTTCGTAGTCTT-3′, bcl-2: forward: 5′-GA ACTGGGGGAGGATTGTGG-3′ and reverse: 5′-GCCGGTwTC AGGTACTCAGTC-3′, bax: forward: 5′-TGAGCGAGTGT CTCAAGCG-3′ and reverse: 5′- CTGCCACTCGGAAAAAG ACC-3′, p53: forward: 5′-GGCCCACTTCACCGTACTAA-3′ and reverse: 5′-GTGGTTTCAAGGCCAGATGT-3′, GAPDH: forward:5′-GCACCGTCAAGGCTGAGAAC-3′ and reverse: 5′-TGGTGAAGACGCCAGTGGA-3′.

### Cell Counting Kit 8 Cell Viability Assay

Cells (5 × 10^4^ cells/well) were seeded in a 96-well plate and grown overnight. Next, cells were treated with vehicle, rotenone (1 μM), and 0–100 ng/ml recombinant human GDF-15 (57-GD-025, R&D Systems, United States). Cell viability was detected using a Cell Counting Kit (CCK) 8 assay (A311-02, Vazyme, Nanjing, China). Briefly, after 24 h, 10 μL of CCK8 solution was added to each well and the incubation continued for another 4 h at 37°C. Absorbance was measured using a multimode microplate reader (Thermo Fisher Scientific Inc., MA, United States) at 450 nm. Cell viability was expressed as a percentage relative to the absorbance of control cells.

### Flow Cytometry

Cell apoptosis induced by rotenone treatment was examined using an Annexin V-PE/7-ADD apoptosis kit (Vazyme, A213) following the manufacturer’s protocol. Briefly, we collected the cells and washed them twice in phosphate-buffered saline. After that, cells were labeled with 500 μL 1 × binding buffer containing 10 μL Annexin V-PE and 10 μL 7-ADD in the dark for 10 min. Subsequently, we used a flow cytometer (BD Falcon; BD Biosciences, Franklin Lakes, NJ, United States) to detect cell apoptosis, and the Annexin V-PE and 7-ADD values were set as the horizontal and vertical axes, respectively, for plot construction. Mechanically damaged, late apoptotic, dual negative/normal, and early apoptotic cells were located in the upper left, upper right, lower left and lower right quadrants of the flow cytometric dot plot, respectively.

### Seahorse XF Cell Mito Stress Test

The mitochondrial functions of cells were measured using a Seahorse XF cell Mito Stress test kit (103015-100, Agilent Technologies, Santa Clara, CA, United States) and a Seahorse XF24 Extracellular Flux Analyzer. To measure the oxygen consumption rate, mitochondrial complex inhibitors (oligomycin [1 μM], FCCP [1 μM], and rotenone/antimycin A [0.5 μM]) were successively added to the cell culture microplate to measure key parameters of mitochondrial function using the Seahorse XF24 Analyzer. Each sample was assayed based on a minimum of three replicates, and the data were normalized to the protein content in each well.

### Terminal dUTP Nick-End Labeling Staining

For apoptosis detection, paraffin-embedded sections were stained using the Terminal dUTP nick-end labeling (TUNEL) technique and an *in situ* apoptosis detection kit (KGA7061, KeyGEN BioTECH, Jiangsu, China) following the manufacturer’s protocols. Briefly, cells were incubated with TUNEL reaction mixture and DAPI for 30 min in a dark room at 37°C after being fixed, blocked, and permeabilized. The images were scanned and analyzed with a fluorescence microscope (Olympus IX73, Japan). The apoptotic ratio was calculated as the ratio of TUNEL-positive cells to total cells.

### Mitochondrial Membrane Potential Measurements

To evaluate the mitochondrial membrane potential (MMP), we used a JC-10 assay kit (ab112134, Abcam). Following the commercial protocols, cells were cultured in a 96-well black plate with a transparent bottom (Costar, Corning, NY, United States). After the experimental procedures, the cell mitochondria were stained with JC-10 under standard conditions for 30 min and kept away from light. Later, the buffer was added to each well, and the plates were read using a fluorescence microplate reader (Spark 10M, TECAN, Zurich, Switzerland). The green fluorescence was read at 490 nm (excitation) and 520 nm (emission). The orange fluorescence was read at 515 nm (excitation) and 590 nm (emission). The data were expressed as the orange/green fluorescence ratio.

### Detection of Reactive Oxygen Species

Treated cells were incubated with DCFH-DA (CA1410, Solarbio, United States) at a concentration of 10 μmol/l for 30 min at 37°C to detect intracellular reactive oxygen species (ROS) levels. Fluorescence was recorded at 488 nm (excitation) and 525 nm (emission) using a flow cytometer (BD Falcon; BD Biosciences, Franklin Lakes, NJ, United States). The mitochondrial ROS levels in different groups of SH-SY5Y cells were determined by performing Mito-Sox staining. Briefly, SH-SY5Y cells were cultured in a fluorescent chamber and subjected to different treatments. Next, SH-SY5Y cells were incubated with 5 μM Mito-Sox (M36008, Invitrogen) for 15 min at 37°C in the dark. The morphology of SH-SY5Y cells was detected with MitoTracker Green FM (Invitrogen, Carlsbad, CA, United States). SH-SY5Y cells subjected to different treatments were incubated for 30 min with DMEM supplemented with 20 nM MitoTracker Green FM. After washing with PBS three times, cells were mounted with DAPI and photographed using a confocal microscope equipped with a camera (Leica DMI8, Germany). At last, the sample was randomly photographed, and the fluorescence intensity was analyzed from five different view fields in each group using ImageJ software based on three independent experiments.

### Statistical Analysis

Statistical analysis was performed using GraphPad Prism 5.01 software (GraphPad, Inc., La Jolla, CA, United States). All analyses were performed depending on the distribution (parametric or non-parametric) of the data. The Shapiro–Wilk’s test for normality was used for this purpose. The results are presented as the mean ± S.E. (standard error). If the data followed a normal distribution, they were analyzed using one-way analysis of variance, whereas two-group comparisons were performed using the Student’s *t*-test. *P*-values < 0.05 were considered statistically significant.

## Results

### Upregulation of GDF15 in Rotenone-Treated SH-SY5Y Cells Reduces Rotenone-Induced Cytotoxicity

**FIGURE 1:**
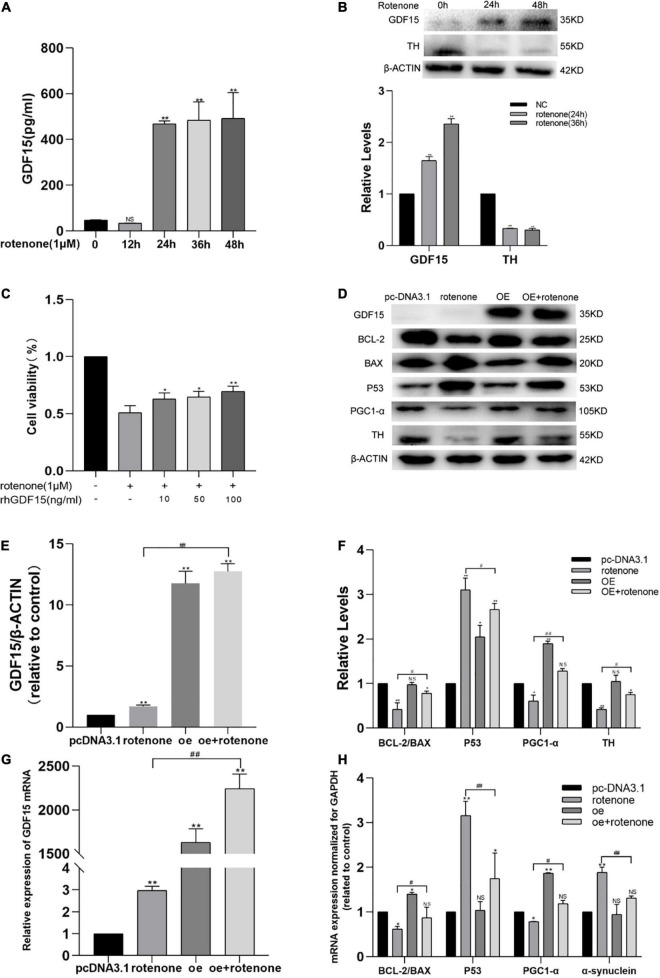
GDF15 expression is elevated in rotenone-treated SH-SY5Y cells and rotenone-induced cellular toxicity is reduced by GDF15. **(A)** The secretion of GDF15 protein was detected with an enzyme-linked immunosorbent assay after cells were exposed to rotenone. The level of GDF15 released showed a time-dependent increase, especially after 24 h. **(B)** Western blotting analysis showing that GDF15 expression was elevated and that tyrosine hydrolase (TH) was decreased in SH-SY5Y cells treated with rotenone for 24 and 36 h. **(C)** Cell viability was detected by a CCK8 assay in SH-SY5Y cells treated with 1 μM rotenone for 24 h and 0–100 ng/mL rhGDF15. **(D–F)** Western blotting analysis of GDF15, Bcl-2/Bax, p53, and PGC-1α in SH-SY5Y cells. β-Actin was used as the internal control. The overexpression of GDF15 had no evident effect on the expression of TH and Bcl-2/Bax under normal conditions. Compared to control group cells, p53 was found significantly increase with reduction of PGC-1α, Bcl-2/Bax and TH in rotenone treated group. And lower expression of p53 was detected in cells with GDF15 overexpression following rotenone treatment compared with rotenone treated-only groups, in addition to evident upregulation of the expression of Bcl-2/Bax, PGC-1α and TH. **(G)** GDF15 mRNA level determined using qRT-PCR. *GDF15* mRNA level in GDF15 overexpression group was higher than that in normal control cell. And compared with rotenone only treated group, the GDF15 mRNA level was also found higher after rotenone treatment in GDF15overexpression cells. **(H)** mRNA expression normalized to that of *GAPDH* measured using PCR. *GDF15* overexpression in SH-SY5Y cells had no evident effect on the expression of α*-syn* under normal conditions, but cell exposure to rotenone further increased the expression of α*-syn* and *p53* and decreased the expression of *Bcl-2/Bax* and *PGC-1*α, whereas *GDF15* overexpression could efficiently alleviate these changes. Data represent the mean ± S.E. from three independent experiments, and differences were analyzed with an unpaired student *t*-test. *P < 0.05, **P < 0.01 as compared to the control group; *#P* < 0.05, *##P* < 0.01, as compared to rotenone-treated only group.

We used a human ELISA kit to measure the secretion levels of endogenous GDF15 protein in the cell culture supernatant following rotenone treatment, and the level of GDF15 released to the presented as a time-dependent increase, especially after 24 h (Figure 1A). In addition, we used western blotting to detect GDF15 and TH expression (Figure 1B). GDF15 concentrations increased significantly (*P* < 0.01) after treatment with rotenone in SH-SY5Y cells, suggesting a time-dependent change. The levels of TH, which is the enzyme that limits the level for dopamine biosynthesis, were downregulated in SH-SY5Y cells treated with rotenone.

To detect the protective effect of GDF15 on rotenone-treated SH-SY5Y cells, a cell viability test was performed using a CCK8 assay in cells treated with different rhGDF15 concentrations (0–100 ng/mL) after exposure to 1 μM rotenone or DMSO for 24 h. Compared with that in control cells (not treated with rotenone or rhGDF15), cell viability was evidently decreased after treatment with 1 μM of rotenone for 24 h, and a concentration-dependent elevation of cell viability was observed after rhGDF15 treatment (Figure 1C). This indicated that rotenone treatment resulted in an elevation of GDF15 expression and rhGDF15 had a protective effect on SH-SY5Y cells treated with rotenone depending on the dose and time.

### Effect of GDF15 on Mitochondrial Protection and Inhibition of Apoptosis in Rotenone-Treated SH-SY5Y Cells

To explore the downstream reactions following treatment with GDF15, the expression levels of several proteins involved in apoptosis were detected using western blotting, and the mRNA levels were quantified using qRT-PCR. Western blotting analyses GDF15 overexpression had no evident effect on the expression of TH and Bcl-2/Bax under normal conditions. It showed that 24-h treatment with 1 μM rotenone causes a marked and significant increase of p53 protein as compared to control group cells, and it causes a decrease of PGC-1α, Bcl-2/Bax, and TH expressions. However, the overexpression of GDF15 proved to attenuate or reduce the effect caused by rotenone treatment (Figures 1D–F). mRNA levels codifying for *Bcl-2/Bax*, *PGC-1*α, *p53*, and α*-syn* were quantified in SH-SY5Y cells by real-time PCR. The overexpression of GDF15 had no evident effect on the expression of α*-syn* under normal conditions. After treatment with rotenone, it could be found that the expression of α*-syn* and *p53* have increased, while *Bcl-2/Bax* and *PGC-1*α was decreased, whereas *GDF15* overexpression could efficiently alleviate these changes (Figures 1G,H). These results indicated that rotenone-induced toxicity might cause apoptosis by regulating Bcl-2/Bax and p53, whereas GDF15 could inhibit this harmful effect.

**FIGURE 2:**
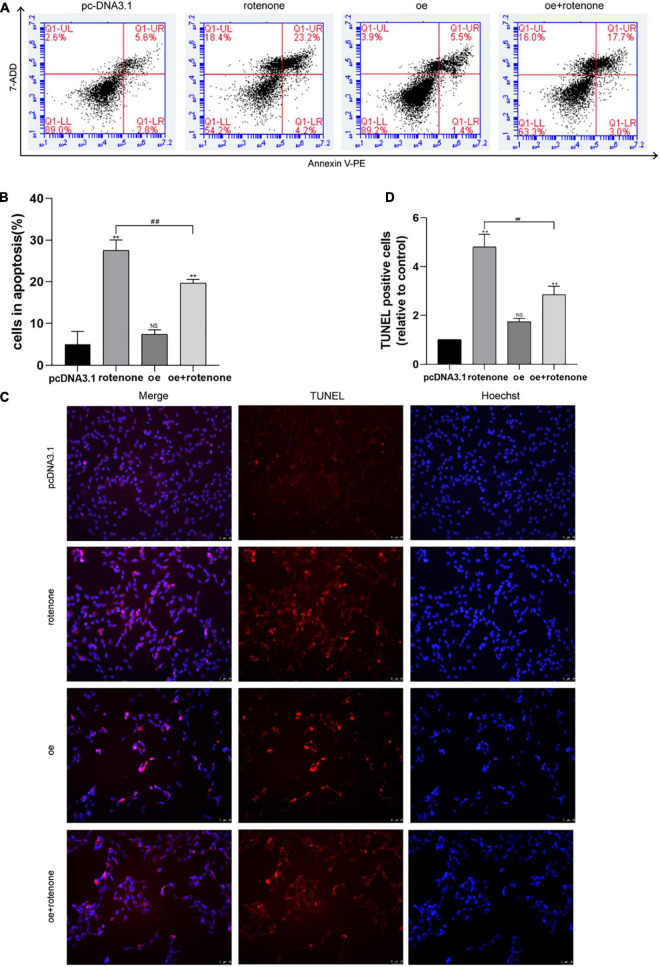
GDF15 decreases rotenone-induced SH-SY5Y cell apoptosis and prevents rotenone-induced mitochondrial damage. **(A,B)** Cell apoptosis was measured by flow cytometry after exposing cells to rotenone for 24 h. Flow cytometry showed an increased percentage of apoptotic cells after treatment with rotenone, whereas cells overexpressing GDF15 showed lower levels of apoptosis in comparison with those in cells only treated with rotenone. **(C,D)** A TUNEL assay was used to examine SH-SY5Y cell apoptosis. Rotenone led to severe SH-SY5Y cell apoptosis compared to that in control and GDF15-overexpressing cells. Data are presented as the mean ± S.E. from three independent experiments, and differences were analyzed with an unpaired student *t*-test. **P < 0.01, as compared to the control group; *##P* < 0.01, as compared to rotenone-treated only group.

Apoptosis was quantified using flow cytometry after exposing cells to rotenone for 24 h. Flow cytometry showed an increased percentage of apoptotic cells after treatment with rotenone (*P* < 0.05), whereas cells overexpressing GDF15 showed lower levels of apoptosis in comparison with cells only treated with rotenone (*P* < 0.05; Figures 2A,B). After rotenone exposure, SH-SY5Y cells exhibited the typical apoptotic characteristics of Hoechst-stained nuclei, including obvious nuclear shrinking with increased density, condensation, and fracture. We further examined SH-SY5Y cell apoptosis by TUNEL assays (Figures 2C,D). The results of TUNEL revealed that rotenone caused the severe apoptosis of SH-SY5Y cells. Although the percent positivity with rotenone increased significantly in comparison with that in the control group (*P* < 0.05), GDF15 overexpression significantly reduced the level of rotenone-induced apoptosis (*P* < 0.05). These results indicated that GDF15 overexpression could suppress cell apoptosis caused by rotenone-induced toxicity.

### GDF15 Prevents Rotenone-Induced Mitochondrial Damage

**FIGURE 3:**
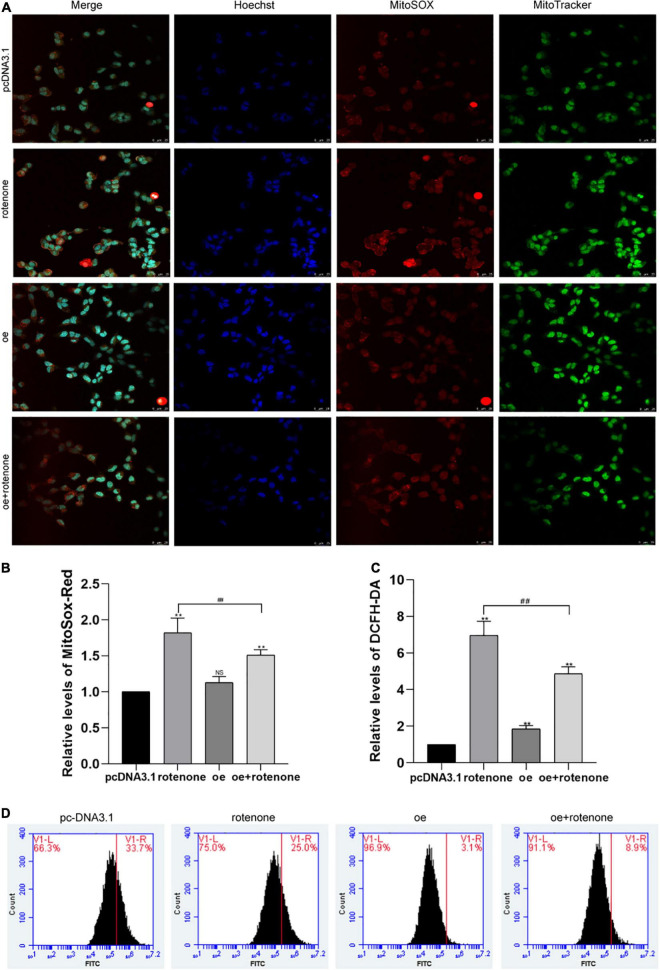
Detection of intracellular and mitochondrial levels of reactive oxygen species (ROS) in SH-SY5Y cells. **(A)** MitoSOX/MitoTracker/Hoechst staining images and fluorescence microscopy were used to determine the level of mitochondrial ROS in rotenone-treated SH-SY5Y cells. Hoechst (blue), Mito-Sox (red), and MitoTracker (green). The ROS level was significantly increased in cells not overexpressing GDF15 compared with that in normal control and GDF15-overexpressing cells. Magnification: 400×. **(B)** Quantification of mitochondrial ROS levels using flow cytometry and Mito-Sox-Red fluorescent probes. **(C,D)** Intracellular ROS generation (%) in SH-SY5Y cells was assessed with the ROS-sensitive fluorometric probe DCFH-DA using flow cytometry. Data are presented as the mean ± S.E. from three independent experiments, and differences were analyzed with an unpaired student *t*-test. **P < 0.01, compared to the control group; *##P* < 0.01, as compared to rotenone-treated only group.

**FIGURE 4:**
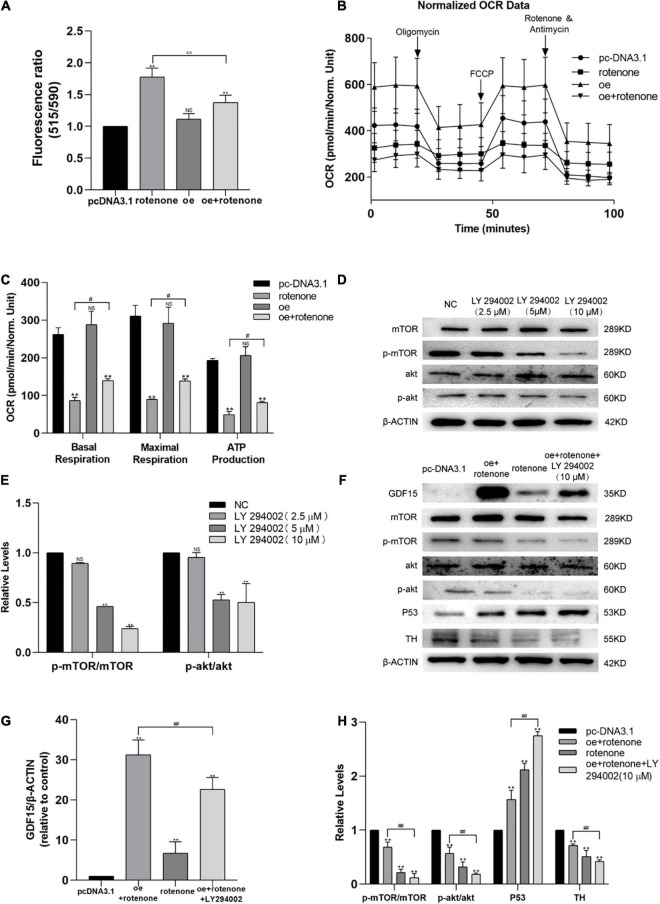
Expression of GDF15 prevents rotenone-induced toxicity with respect to the mitochondrial membrane potential (MMP) and might require Akt/mTOR-dependent phosphorylation. **(A)** MMP in SH-SY5Y cells following treatment with rotenone. The MMP, measured using a JC-10 assay, was significantly decreased by rotenone. **(B,C)** The oxygen consumption rate (OCR) of SH-SY5Y cells was measured using a Seahorse XF24 Extracellular Flux Analyzer. Basal respiration, maximal respiration, and ATP production in SH-SY5Y cells treated with rotenone were significantly lower than those in control and GDF15-overexpressing cells. Also, when compared to rotenone group, it could be found that basal respiration, maximal respiration, and ATP production improve in rotenone treated GDF15 overexpression group. **(D,E)** The levels of phosphorylation of both Akt and mTOR in SH-SY5Y cells after 3 h treated with various concentrations of the PI3K/Akt specific inhibitor LY294002 (0–10 μM) were assessed by western blotting analysis. **(F–H)** Western blotting showing increased GDF15 expression after transfection with the GDF15-overexpression plasmid, which increased the phosphorylation of both Akt and mTOR. Inhibition of the PI3K/AKT pathway using LY294002 upregulated the expression levels of p53 and eliminated the protective effect of GDF15. Anti-β-actin was used as an internal control. Data are presented as the mean ± S.E. (standard error) from three independent experiments, and differences were analyzed with an unpaired student *t*-test. **P < 0.01, compared to the control group; *#P* < 0.05, *##P* < 0.01, as compared to rotenone-treated only group or rotenone-treated group after transfection with the GDF15 overexpression plasmid.

DCFH-DA and Mito-Sox were used to detect the level of intracellular ROS and mitochondrial ROS, respectively, in different groups of SH-SY5Y cells. As illustrated in Figures 3A–D, the ROS level was significantly increased in cells not overexpressing GDF15 compared with that in normal control and GDF15-overexpressing cells. Collectively, our results reveal that GDF15 reduces SH-SY5Y cell apoptosis by decreasing ROS generation. MMP was assessed by JC-10 assays, and this was significantly decreased by rotenone treatment, whereas GDF15 could mitigate the toxic effect of rotenone (Figure 4A). These results showed that GDF15 overexpression could prevent rotenone-induced mitochondrial damage.

The mitochondrial function of SH-SY5Y cells was evaluated by measuring the oxygen consumption rate using a Seahorse XF24 Extracellular Flux Analyzer. The oxygen consumption rates of rotenone-treated SH-SY5Y cells were significantly lower than those of control and GDF15-overexpressing cells, as evidenced by the basal respiration, maximal respiration, and ATP production. And the level of basal respiration, maximal respiration, and ATP production was found an improvement in rotenone treated GDF15 overexpression group compared with rotenone group (Figures 4B,C). These data suggested that GDF15 impairs oxidative phosphorylation and mitochondrial function.

### GDF15 Expression Requires Akt/mTOR-Dependent Phosphorylation

SH-SY5Y cells were treated with thee PI3K/Akt-specific inhibitor LY294002 (10 μM) 3 h before transfection with the GDF15 overexpression plasmid according to western blotting analysis (Figures 4D,E). As shown in Figures 4F–H, increased GDF15 expression after transfection with the GDF15 overexpression plasmid increased the phosphorylation of both Akt and mTOR. Moreover, inhibition of the PI3K/AKT pathway using LY294002 upregulated the expression levels of p53 and eliminated the protective effect of GDF15, which suggested that the effect of GDF15 expression was related to Akt/mTOR-dependent phosphorylation.

## Discussion

The present study aimed to investigate the association between endogenous GDF15 and mitochondrial function in PD. Our research and other former investigations have proved that the serum GDF15 level in PD patients is different compared to that in control individuals (Yao et al., 2017). The significance of GDF15 has been demonstrated in a model of damage to the central and peripheral nervous system (Schober et al., 2001; Strelau et al., 2009; Schindowski et al., 2011; Wang et al., 2015), with local supplementation with GDF15 resulting in an improvement in axonal and functional regeneration of dorsal root ganglion neurons (Mensching et al., 2012). Furthermore, it has been reported that GDF15 is expressed in the choroid plexus and acts as an important neurotrophic factor for motor and sensory neurons in the central nervous system (Strelau et al., 2009; Montero et al., 2016). In this study, we used a CCK-8 assay to detect cell viability, and the results demonstrated that the overexpression of GDF15 significantly increased SH-SY5Y cell viability and suppressed the effect of oligomycin on SH-SY5Y cells.

In the current study, we show that rotenone-induced mitochondrial dysfunction and apoptosis are less severe in GDF15-treated SH-SY5Y cells, which suggests a causal relationship between GDF15 and mitochondrial dysfunction. Our results including flow cytometric and TUNEL assay results showed that GDF15 expression reduces apoptosis in SH-SY5Y cells and indicated that GDF15-overexpressing cells and GDF15-treated cells show less damage after treatment with rotenone, whereas the level of p53 was lower and that of PGC1α was higher. These data demonstrate that GDF15 is a positive regulator in SH-SY5Y cells treated with rotenone. Numerous studies among humans have reported that the activity of GDF15 is upregulated under stressful circumstances as a response to tissue insults. Among healthy people, the level of serum GDF15 is very low; in contrast, the plasma GDF15 levels are significantly higher in the elderly and in patients with pathological conditions, such as coronary vascular diseases, diabetes mellitus, neurological degeneration, and cancer (Fujita et al., 2016; Conte et al., 2019). We inferred that GDF15 inhibits the expression of p53 and interacts with Bcl-2/Bax to regulate mitochondrial function and apoptosis.

In this study, we used seahorse to measure mitochondrial function and JC-10 to detect the MMP. Our results revealed that GDF15 overexpression could dramatically reverse the rate of oxygen consumption in SH-SY5Y cells. The JC-10 assay showed that rotenone significantly decreased the MMP, whereas GDF15 could interrupt the toxic effect of rotenone. The relationship between PD and mitochondrial function has attracted increasing attention recently. Some researchers have described the pathological conditions of mitochondria as a potential mechanism of PD (Keane et al., 2011; Grünewald et al., 2016). Several genes associated with familial PD, such as *PARK2* and *PINK1*, are directly involved in processes like mitophagy, which maintain mitochondrial health. Furthermore, it has been proven that a *VPS35* mutant reduces the MMP and impairs PINK1/Parkin-mediated mitophagy in PD (Ma et al., 2021). Recently, some scientists have developed induced pluripotent stem cell (iPSC)-derived astrocytes that transferred functional mitochondria to rescue injured DA neurons (derived from iPSCs) after exposure to rotenone in an *in vitro* PD model (Cheng et al., 2020). Some researchers hypothesized that GDF15 might be helpful for mitochondrial diseases (Fujita et al., 2015; Yatsuga et al., 2015). Therefore, we presumed that GDF15 might protect mitochondrial function by regulating the MMP and oxygen consumption of SH-SY5Y cells.

GDF15 has been identified by different groups using several cloning strategies (Baek and Eling, 2006). Lately, based on the crystal structures of mature GDF15 and the mature GDF15–glial cell-derived neurotrophic factor (GDNF) receptor alpha-like (GFRAL) extracellular domain complex, it has been found that GDF15 belongs to the family of glial cell-derived neurotropic factors, with activity dependent on RET but not on TGF-β receptors (Hsu et al., 2017). The promoter of *GDF15* in humans has been characterized and has binding sites for a number of transcriptional factors, including p53, EGR-1, CREB, Sp1, CHOP, ER stress, and ATF3. The expression GDF15 is also increasing by peroxisome proliferator-activated receptor (PPAR) γ ligands (Baek et al., 2003; Chintharlapalli et al., 2005; Yamaguchi et al., 2008) and PI3K/AKT/GSK-3β pathways (Baek et al., 2004; Yamaguchi et al., 2004). However, the pleiotropic effects of GDF15 have been shown to depend on the cell type and micro-environment in which the cell is located (Hromas et al., 1997; Duong et al., 2008; Ding et al., 2009).

Mitochondria play an important role in neurodegeneration and neuroprotection. Mitochondrial biogenesis is a process whereby new mitochondria are generated from existing mitochondria, regulated by PGC-1α (Uittenbogaard and Chiaramello, 2014). Some researchers hypothesized that PGC-1α might be upregulated by Akt-activated CREB, leading to the induction of mitochondrial biogenesis (Li et al., 2017). According to previous reports, GDF15 is suggested to have a protective role against different insults via PI3K–Akt signaling pathways (Xu et al., 2006; Kempf et al., 2011; Kalli et al., 2019; Tarfiei et al., 2019). Cao and co-workers reported that amentoflavone protects dopaminergic neurons in methyl-4-phenyl-1,2,3,6-tetrahydropyridine-induced PD-model mice via PI3K-Akt signaling pathways (Cao et al., 2017). In our study, we tried to identify the regulatory pathway that modulates GDF15. Our results showed that GDF15 overexpression was observed after SH-SY5Y cells were exposed to rotenone, which increased the phosphorylation levels of mTOR and Akt, indicating the activation of Akt/mTOR. Moreover, inhibition of the PI3K/AKT pathway using LY294002 upregulated the expression levels of p53 and eliminated the protective effect of GDF15. According to previous studies, GDF15 might activate PI3K/Akt signaling pathways and finally promote cell proliferation in human cervical cancer (Li et al., 2018). Thus, we assumed that GDF15 might participate in metabolic processes in SH-SY5Y cells by inducing Akt/mTOR signaling pathways.

## Conclusion

Our investigation indicates a putative role for GDF15 in mitochondrial function in rotenone-treated SH-SY5Y cells. GDF15 can regulate PGC1α by influencing p53, thus protecting mitochondrial function and inhibiting apoptosis in rotenone-treated SH-SY5Y cells. Moreover, these effects might be related to its anti-apoptotic effects mediated by activation of the PI3K/Akt/mTOR pathway.

## Data Availability Statement

The raw data supporting the conclusions of this article will be made available by the authors, without undue reservation.

## Author Contributions

PZL and HL designed the study with the help from CS and YL. PFL, CS, and YL supervised the project. BZ, XZ, and RD helped PZL complete the study search. PZL and HL performed all analysis and wrote the manuscript. All authors discussed and contributed to the final manuscript.

## Conflict of Interest

The authors declare that the research was conducted in the absence of any commercial or financial relationships that could be construed as a potential conflict of interest.

## Publisher’s Note

All claims expressed in this article are solely those of the authors and do not necessarily represent those of their affiliated organizations, or those of the publisher, the editors and the reviewers. Any product that may be evaluated in this article, or claim that may be made by its manufacturer, is not guaranteed or endorsed by the publisher.
